# The alternative model of silicone for experimental simulation of suture of living tissue in the teaching of surgical technique^1^

**DOI:** 10.1590/s0102-865020190040000010

**Published:** 2019-04-29

**Authors:** Ana Paula Gurjão da Silva, Juan Eduardo Rios Rodriguez, Maria Conceição de Oliveira, Róbson Miguel de Araújo Negreiros, Leonardo Pessoa Cavalcante

**Affiliations:** I Graduate student, Laboratory of Surgical Technique and Experimental Surgery, Medical School, Universidade Federal do Amazonas (UFAM), Manaus-AM, Brazil. Conception, design and scientific content of the study; acquisition and interpretation of data; manuscript preparation.; II PhD, Medical School, UFAM, Manaus-AM, Brazil. Conception, design and scientific content of the study; critical revision, final approval.; III MSc, Laboratory of Surgical Technique and Experimental Surgery, Medical School, UFAM, Manaus-AM, Brazil. Conception, design and scientific content of the study; critical revision, final approval.; IV PhD, Laboratory of Surgical Technique and Experimental Surgery, Medical School, UFAM, Manaus-AM, Brazil. Conception, design and scientific content of the study; critical revision, final approval.

**Keywords:** Education, Medical, Teaching, Sutures, General Surgery

## Abstract

**Purpose:**

To develop a silicone alternative model of tissue suture simulation to be used in the teaching of surgical technique.

**Methods:**

Twelve alternative models of silicone for tissue suture simulation were manufactured and implemented as a tool for suture pattern training of undergraduate medical students of Universidade Federal do Amazonas. Forty-eight students participated in the research. The evaluation of the proposed model was done through a questionnaire using the Likert scale, in order to verify the student satisfaction index of the alternative resource and its performance as opposed to the model historically used in the discipline, which is to suture in cloths.

**Results:**

The alternative model showed satisfactory results, especially with respect to the structural aspect, such as, better perception of anatomical planes, handling and transport. About 89.58% of positive concordant responses demonstrating expressive approval for incorporation of a complementary form of the alternative methodological proposal of the discipline of surgical technique.

**Conclusions:**

The model developed for experimental simulation of tissue sutures has proved to be a fully feasible alternative method for the training of this surgical skill. It is a simple, reproducible and low-cost model.

## Introduction

 The learning and improvement of basic surgical techniques constitutes one of the pillars of medical training. Historically, teaching of the operative technique assumed different perspectives, according to the availability of resources and with the scientific and behavioral human advances. It is worth to point that, regardless of the type of resource used, the development and the constant execution of surgical skills are fundamental for acquisition of skills during medical graduation^1^ ^,^ ^2^.

 Over many years, teaching of surgical technique was primarily exercised through experimental surgery in animals. Despite the excellence in teaching/learning with the use of this resource, ethical, legal and humanitarian implications related to the use of animals in education, led to a drawback regarding the acceptance of this method, especially since the emergence of the 3R’s^3^ guidelines, which advocates reduction, refinement and replacement of the use of animals whenever possible, especially in a teaching scenario.

 Thus, alternatives have been sought to replace the use of animals for the teaching of basic surgical techniques. There has been a constant search for models that are closer to the reality of biological material and that can guarantee psychomotor skills for performing basic surgical procedures. Simulation of surgical synthesis (tissue suture), a fundamental time for the regeneration of incised or traumatized tissues, plays a prominent role in the teaching of operative technique, since it requires more time of practice and dedication for its assimilation by the medical student. However, contrary to their importance, alternative models of surgical synthesis are particularly poorly described in the literature and, when present, these models tend to be costly^4^. 

 Therefore, the objective of this study was to construct a simple and inexpensive model that would mimic the anatomical planes of the human tissue, made of silicone, to be used in the training of suture patterns and to compare it to the method currently used for the teaching of suture patterns. 

## Methods

### Study design

 The research began after approval by the Research Ethics Committee, Universidade Federal do Amazonas.

 It was an experimental and descriptive research, performed in the Laboratory of Surgical Technique and Experimental Surgery, Medical School, UFAM. The participants of the study were the undergraduate medical students who attended the laboratory in the second semester of 2017 and the first semester of 2018 and that formally agreed to participate in the study. It should be emphasized that the participation had no influence on the evaluations of the course, and that the identity of participants was ensured in all steps of the research. In total, there were 48 participants. 

### Making the alternative model for suture training

**Figure 1:**
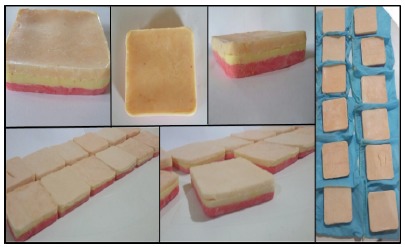
Final aspect of the alternative models made with silicone.

**Figure 2:**
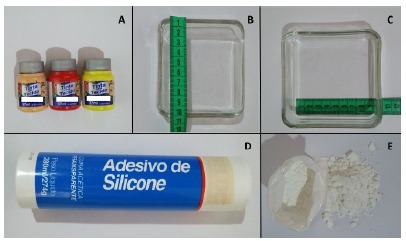
Materials used in the design of alternative models. (**A**) Fabric paints, (**B**) and (**C**) Mold, (**D**) Transparent silicone sealant, (**E**) Corn starch.

 Twelve molds of size 10 x 9cm (Fig. 1) were made of silicone layers of different thicknesses, mimetizing the layers of the skin. As a result of the texture of the material, we sought to mimic the suture sensation in living tissue. For this purpose, the following materials were used: transparent silicone sealant, cornstarch and fabric paints of three different colors, as well as a plastic or wooden box (10 x 9 cm) for mold making (Fig. 2). The average time used to make each model was between 15 to 20 minutes, and it consisted in the accomplishment of the following steps:

1) Mixing approximately 30g of cornstarch with 15g of silicone-based sealant;
2) The resulting blend dyed with the color paint corresponding to each mimicked layer of living tissue (skin, subcutaneous cellular tissue and muscle layer);
3) The mass resulting from step 2 placed into the 10 x 9 cm mold to acquire the shape of the model;
4) The first 3 steps were repeated to construct each layer individually, which were placed overlap one another to form the 3-layer model.

### Application of the alternative model

 The application of the alternative model in the first phase of the research (semester 2017/2) and in the second phase (semester 2018/1) consisted of execution of interrupted sutures (single, X, U vertical and U horizontal stiches) and continuous suture (simple running suture) using the historical model of teaching used the laboratory (cloths) and the alternative developed model. The sutures were made with nylon thread, 3-0 thick, with a 3 cm triangular curved (3/8) needle.

 The historical method of suture training in the laboratory consists in the practice of suturing in cloth, in which spaces are created to mimic operative wounds. This was the model used for comparison against the model that was developed.

### Form of evaluation of the alternative model

 Taking into consideration the fact that a model for simulated training in surgical skills has limited value when its target public does not accept it, we chose the evaluation of the proposed model through a questionnaire to be answered individually by the participants after training with the 2 models. It is a subjective questionnaire using the Likert scale^5^ (Chart 1), intending to verify the satisfaction index of students in relation to the alternative resource and its performance as opposed to the historical model.

**Chart 1:** Likert-style questionnaire**.**

Questions	Grading				
	0	1	2	3	4
Q1. Is there a need to develop new alternative methods of teaching instead of using animals in teaching surgical techniques?	Strongly disagree	Partially disagree	Do not agree or disagree	Partially agree	Strongly agree
Q2. Can interrupted suture patterns be performed with this alternative silicone model?					
Q3. Can continuous suture patterns be performed with this alternative silicone model?					
Q4. Is this alternative model appropriate for the development of surgical skills, repetition of procedures, and association of theory with practice?					
Q5. Does the size and weight of this alternative model make it easy to handle and transport?					
Q6. Does the use of this alternative model allow the assimilation of other concepts important for the development of surgical skills, such as the choice and manipulation of surgical instruments?					
Q7. Structurally, does the silicone model allow for a better spatial perception of the anatomical planes of the skin compared to training in cloths?					
Q8. With this silicone model is it possible to develop the dexterity necessary for the synthesis (suture) of tissues?					
Q9. Does silicon model training, compared to suture training in cloths, ensure greater confidence when you perform the same procedure on an actual patient?					
Q10. Is performing sutures in the alternative silicone model better than performing sutures on the cloth?					
Q11. Should the suture training in the silicone model be added to the training performed on the cloth?					
Q12. In your opinion, should the suture training on the silicone model replace the training performed on the cloth?			Yes		
			No		

### Analysis

 Since it was a descriptive study and categorical type variables, data were analyzed using absolute and relative frequency for descriptive statistical analysis according to the participants’ expressed opinion. The data were collected in a standard form, tabulated and analyzed using the EI-Epi-Info 7.2.2.2 Program and, once organized, all the data obtained were submitted to a detailed content analysis.

## Results

**Figure 3:**
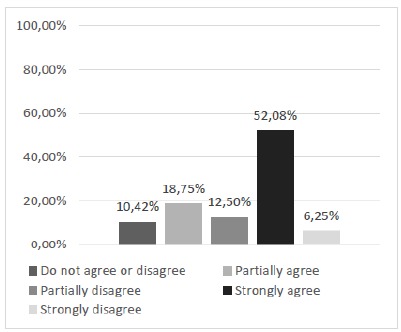
Is there a need to develop new alternative methods to replace the use of animals in teaching the surgical technique?

 The first point raised by the questionnaire sought to highlight the participants’ perception of the need to develop new alternative models to replace the use of animals in the learning of surgical techniques. The descriptive analysis using the relative and absolute frequency calculation showed that 70.83% of the participants declared positive opinions of partial or total agreement with the questioning (Fig. 3). Subsequent questions, specifically the range of questions 2 to 8, had, as main purpose, the subjective evaluation of the model regarding its physical characteristics and its performance in the training of skills of basic surgical techniques, specifically the suture. 

**Figure 4:**
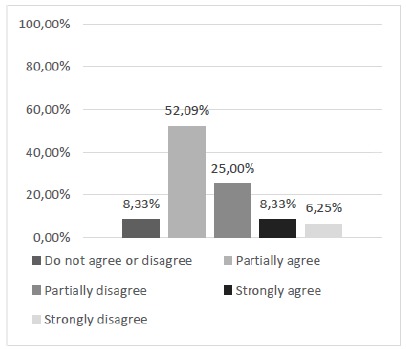
Can discontinuous suture patterns be performed with this alternative silicone model?

**Figure 5:**
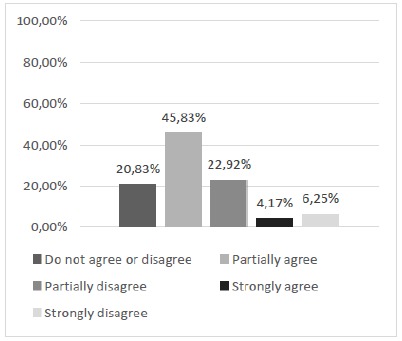
Can continuous suture patterns be performed with this alternative silicone model?

 Thus, questions 2 and 3, in turn, that dealt with the feasibility of discontinuous and continuous suture patterns were obtained satisfactorily under the appreciation of the majority of the study participants (Figs. 4 and 5). Regarding the achievement of continuous suture patterns (Question 3), although the majority manifested positively, there was a significant joint percentage (43.75%) of manifestations of partial disagreement and neutrality.

**Figure 6:**
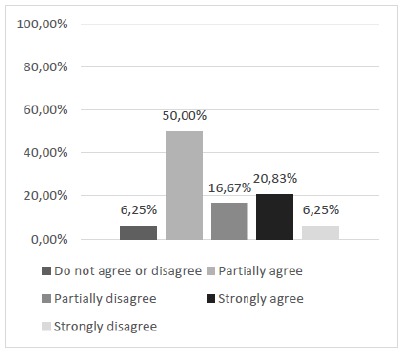
Is this alternative model appropriate for the development of surgical skills, repetition of procedures, and association of theory with practice?

 Regarding the adequacy of the alternative model for the development of surgical skills, repetition of procedures and association of theory with practice (question 4), the analysis showed that approximately 71% of participants attested for a positive opinion considering the sum of partial and total agreement for this evaluation question (Fig. 6).

**Figure 7:**
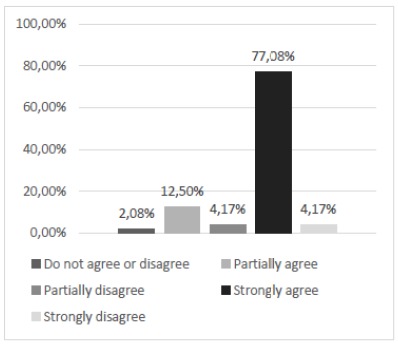
Does the size and weight of this alternative model make it easy to handle and transport?

 The evaluation of the participants’ satisfaction regarding the structural aspect of the model, through questioning about the ease of transportation and handling, taking into account the weight and dimensions of the part (question 5) showed a relative frequency of 77.08% for the total agreement in this question (Fig. 7). In view of the result, we evaluated that in relation to the structural aspect, the alternative model reached a high degree of acceptance on the part of the students participating in the research, a fact of significant importance for the integral receptivity of the model and, in addition, demonstrates the accessibility of the same for the training in different environments.

**Figure 8:**
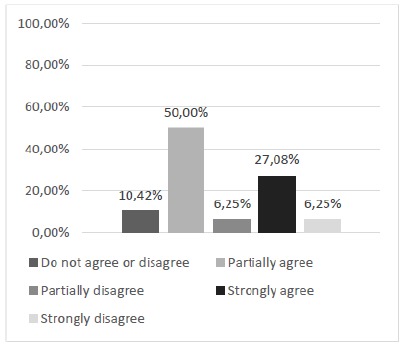
Does the use of this alternative model allow the assimilation of other concepts important for the development of surgical skills, such as the choice and manipulation of instruments?

 About the possibility of assimilation of other concepts important for the development of surgical skills, for example, choice and manipulation of instruments (question 6), we note a significant satisfaction of the participants regarding this interconnection of concepts with the use of the model alternative (Fig. 8).

**Figure 9:**
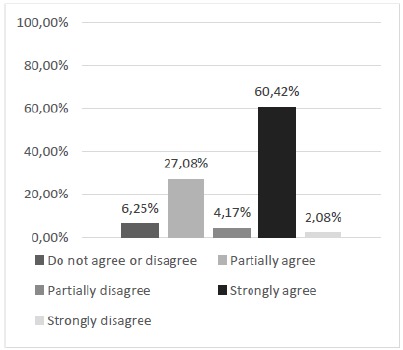
Structurally, does the silicone model allow for a better spatial perception of the anatomical planes of the skin compared to training in cloths?

 The analysis of the participants’ satisfaction regarding the spatial perception of the anatomic planes in the alternative model in comparison to the suture training in the cloth (question 7) showed a sum of total and partial agreement of 87%, indicating a given good perception from the comparative point of view between the two forms of training (Fig. 9).

**Figure 10:**
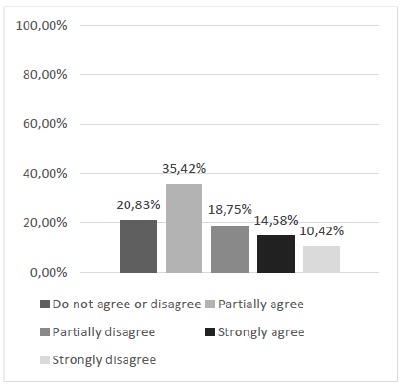
With this silicone model, is it possible to develop the dexterity necessary for the synthesis (suture) of tissues?

 Regarding the development of surgical dexterity for tissue synthesis with the use of the alternative model, there were widely divergent opinions, mostly pointing to a neutral opinion and partial disagreement, probably indicating that the development of dexterity involves many other aspects, and do not depend solely on the training model (Fig. 10).

**Figure 11:**
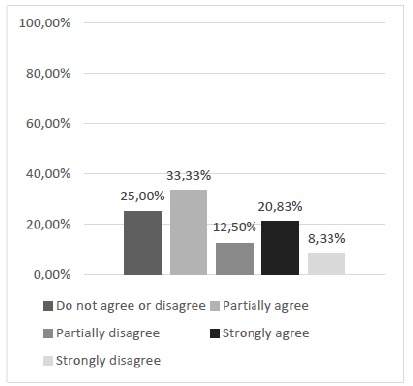
Does silicon model training, compared to suture training in wipes, ensure greater confidence when you perform the same procedure on an actual patient?

 The final questions (9, 10 and 11) represented the essentially comparative questions between the current cloth suture model and the alternative model. Question 9 brought the following question to survey participants: “Does training with the silicone model, compared to suture training in wipes, ensure greater confidence when you perform the same procedure on a real patient? “The results obtained from the analysis of the relative and absolute frequency (Fig. 11) showed well-divided opinions, but with a predominance of positive ones indicating, in this sense, that the acquisition of confidence for the accomplishment of suture in vivo does not depend on the training method employed. 

**Figure 12:**
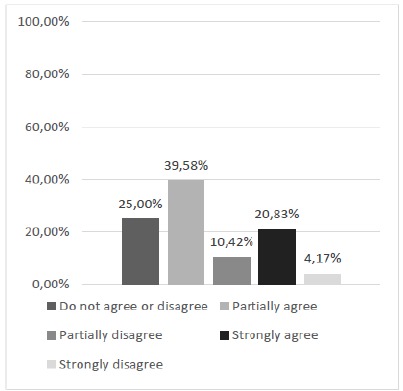
Student opinion about the superiority of one model compared to the traditional model, suture in cloth.

 In order to evaluate the similarity or superiority between the models, the following question was asked: Is suture training in the alternative silicone model better than the to suture using cloth? The result provided important information in order to define the students’ satisfaction regarding the use of the alternative method (Fig. 12).

**Figure 13:**
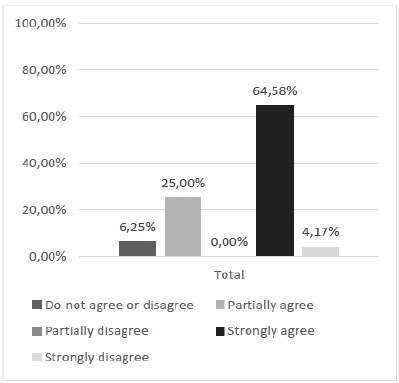
The training of sutures in the silicone model should be associated with the training performed on the cloth.

**Figure 14:**
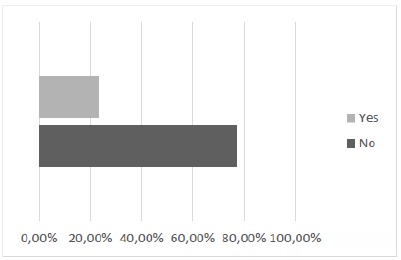
Should the suture training on the silicone model replace the training performed on the cloth?

 Accordingly, it was also sought to verify the opinion of the students regarding the possibility of association of the two methods (alternative and current) for the training of sutures. The result showed 89.58% of positive concordant responses demonstrating expressive approval for incorporation of a complementary form of the alternative methodological proposal in the practical classes of the TOCE discipline (Fig. 13). And finally, regarding the opinion of the possible replacement of the current method by the alternative method, our results indicate a contrary majority opinion to the substitution of one method by the other (Fig. 14).

## Discussion

 Our results allows us to infer that, from the medical students’ point of view, there is an insufficiency of new alternative models for the training of surgical skills, and, therefore, points to the need of their development. Current studies indicate that cheap and viable alternatives to traditional teaching methods should always be sought^2^ ^,^ ^6^ ^,^ ^7^. Many of these studies have been developed to present alternative models for the practice of surgical techniques, mainly aimed at suture training, since, following the expectation, the demand for these models should increase^6^.

 As observed in the results, the patterns of discontinuous and continuous sutures are feasible to be performed with the alternative model, with some proviso for the accomplishment of continuous sutures demonstrating, in this sense, possibly some dissatisfaction as to the feasibility of accomplishment of these patterns and the necessity of improvement of the model for this issue.

 Regarding the structural aspect of the model, especially related to its handling and transportation, a very significant result was found. According to other literature on the theme, Denadai^1^, the choice of the alternative model should not be based solely on its fidelity, but should be based on other aspects such as versatility, storage, availability, ease of transportation and others^1^. Thus, as evidenced in other models, the ease of transportation is an important aspect to be considered, since it allows the training in multiple environments, further reinforcing the acquisition and improvement of skills^4^.

 The results showed that silicone could adequately mimic the spatial representation of anatomical planes, indicating that silicone is a material that presents excellent properties since it is a substance that has the possibility of being textured and dyed to mimic the reality of the tissue^6^.

 In relation to the students’ perception regarding the acquisition of confidence in the accomplishment of suture in vivo, our results point out that the transfer of abilities to the real environment is independent of the level of fidelity of the model used, as highlighted in other literature on the subject. Some studies also emphasize that the use of low-cost models can be a good opportunity for practice without compromising the acquisition of skills and safety to perform suturing in a real patient^2^ ^,^ ^7^ ^,^ ^10^.

 The very expressive opinion of the students to not to replace the traditional model, but to associate both, can indicate that the training under various conditions, based on the complementarity of methods and learning, results in the acquisition of skills in a more satisfactory way, as described in some studies addressing this issue^4^.

### Study limitations

 It is important to recognize that the present study has some limitations, among which we can mention the absence of an objective evaluation of the skills acquisition and technical performance of the research participants, besides the absence of evaluation of the technical results of the alternative model related to aspects such as resistance, limitation of use and durability, and finally the lack of a targeted and specific questionnaire to the traditional model (cloth suture) for better comparability of the models. Moreover, we believe that there may have been a bias of agreement, since the observer was present during the data collection to assist in completing the questionnaire. Therefore, new research should be done using comparative and long-term modeling.

## Conclusion

 The model for experimental simulation of sutures proved to be a simple, reproducible and viable alternative for the training of surgical skills.
